# Green Apple e-Cigarette Flavorant Farnesene Triggers Reward-Related Behavior by Promoting High-Sensitivity nAChRs in the Ventral Tegmental Area

**DOI:** 10.1523/ENEURO.0172-20.2020

**Published:** 2020-08-11

**Authors:** Skylar Y. Cooper, Austin T. Akers, Brandon J. Henderson

**Affiliations:** Department of Biomedical Sciences, Marshall University, Joan C Edwards School of Medicine, Huntington, WV 25703

**Keywords:** electrophysiology, flavorants, microscopy, nicotinic receptors, reward-related behavior

## Abstract

While combustible cigarette smoking has declined, the use of electronic nicotine delivery systems (ENDS) has increased. ENDS are popular among adolescents, and chemical flavorants are an increasing concern because of the growing use of zero-nicotine flavored e-liquids. Despite this, little is known regarding the effects of ENDS flavorants on vaping-related behavior. Following previous studies demonstrating the green apple flavorant, farnesol, enhances nicotine reward and exhibits rewarding properties without nicotine, this work focuses on the green apple flavorant, farnesene, for its impact on vaping-related behaviors. Using adult C57BL/6J mice, genetically modified to contain fluorescent nicotinic acetylcholine receptors (nAChRs), and farnesene doses of 0.1, 1.0, and 10 mg/kg, we observed farnesene-alone produces reward-related behavior in both male and female mice. We then performed whole-cell patch-clamp electrophysiology and observed farnesene-induced inward currents in ventral tegmental area (VTA) putative dopamine (pDA) neurons that were blocked by the nAChR antagonist, DhβE. While the amplitudes of farnesene-induced currents are ∼30% of nicotine’s efficacy, this indicates the potential for some ENDS flavorants to stimulate nAChR function. Additionally, farnesene enhances nicotine’s potency for activating nAChRs on VTA dopamine neurons. This may be because of changes in nAChR stoichiometry as our data suggest a shift toward high-sensitivity α4β2 nAChRs. Consequently, these data show that the green apple flavorant, farnesene, causes reward-related behavior without nicotine through changes in nAChR stoichiometry that results in an enhanced effect of nicotine on VTA dopamine neurons. These results demonstrate the importance of future investigations into ENDS flavorants and their effects on vaping-related behaviors.

## Significance Statement

Although combustible cigarette use has decreased by ∼11% in America over the past two decades, the use of electronic nicotine delivery systems (ENDS) has increased by 135% and 218% among high school and middle school students, respectively, in the last two years alone (Cullen et al., 2018, 2019). Because of the fact that most ENDS users vape flavored nicotine products and the growing use of zero-nicotine flavored e-liquids, it raises the questions of how chemical flavorants alter nicotine addiction and if they increase abuse liability themselves. We show that one chemical flavorant and odorant of green apple, farnesene, causes reward-related behavior on its own. These results increase our understanding on how flavorants promote neurologic changes and affect nicotine addiction.

## Introduction

Tobacco use remains the leading preventable cause of disease and death in America and results in nearly half a million deaths per year. Although there has been a decline in the use of combustible cigarettes, the use of electronic nicotine delivery systems (ENDS) has increased tremendously with over three million users between the ages of 14 and 18 (Cullen et al., 2018). The former United States Food and Drug Administration (FDA) Commissioner, Scott Gottlieb, stated that he believes the ENDS companies are creating a new demographic market among the youth, rather than simply helping adult smokers quit; which was the original objective for the production of ENDS (FDA, 2018). Since their inception, ENDS have become more of a concern among the adolescent population because of the dramatic increase in use among their age demographic and because of the numerous flavor options available (Cullen et al., 2018; FDA, 2018; Schneller et al., 2018; Mead et al., 2019). Unlike combustible cigarettes which are limited to menthol flavor, there is no restriction on flavored ENDS and currently over 15,000 unique flavors are on the market (Hsu et al., 2018). Additionally, >90% of adolescent and ∼70% of adult ENDS users prefer flavored products (Schneller et al., 2018; Mead et al., 2019).

Menthol is the most prominent flavor for tobacco products and was considered to be an inert flavor additive, yet, studies have shown that menthol enhances nicotine reward-related and reinforcement-related behaviors in rodents (Wang et al., 2014; Biswas et al., 2016; Henderson et al., 2017). The menthol-induced enhancement is because of a combination of effects on nicotinic acetylcholine receptor (nAChR) upregulation (Brody et al., 2013; Henderson et al., 2016, 2017), dopamine neuron excitability (Henderson et al., 2018), dopamine release (Zhang et al., 2018), and TrpM8-dependent mechanisms (Fan et al., 2016). In addition to menthol, similar investigations into other popular ENDS flavors are being conducted. A recent report determined that green apple and other fruit flavors are nearly the most popular among all flavorant options available (Espino-Díaz et al., 2016; Omaiye et al., 2019). An investigation into one chemical flavorant of green apple, farnesol, reported that farnesol produced reward-related behavior in mice in the absence of nicotine (Avelar et al., 2019). Furthermore, this effect was sex-dependent and was found to be caused by changes in nAChR upregulation and ventral tegmental area (VTA) dopamine neuron firing in males only at the doses examined.

Based on these previous investigations, we have examined another chemical flavorant of green apple, farnesene, to determine its effect on vaping-related behaviors. We used conditioned place preference (CPP) assays to observe farnesene’s effect on reward-related behavior, confocal microscopy to observe nAChR changes on midbrain dopamine and GABA neurons associated with the reward pathway, and whole-cell patch-clamp electrophysiology to study changes in midbrain neuron function. These experiments were performed with mice genetically modified to express fluorescently labeled nAChRs and were used in microscopy or electrophysiology following behavioral assays. Following these experiments, we have demonstrated that farnesene enhances nicotine reward-related behavior and prompts reward-related behavior in the absence of nicotine, and thus, may explain the prominence of green apple flavors among ENDS users. Additionally, we identified farnesene acts as a partial agonist for nAChRs and stimulates inward currents on VTA dopamine neurons. Finally, we observed that while farnesene does not exert a pronounced effect on VTA nAChR upregulation, long-term treatment with farnesene alters nAChR stoichiometry to promote the assembly of high-sensitivity nAChRs. Overall, these data identify the significance in studying ENDS flavors and demonstrate the potential underlying mechanisms that may promote the initiation and maintenance of ENDS use among the youth.

## Materials and Methods

### Reagents and dose selection

(–)-Nicotine hydrogen tartrate (product number 1463304) and farnesene (product number W383902-100G-K, lot number MKCB6021) were obtained from Sigma-Aldrich. We determined the relevant dose of farnesene in regard to mouse behavioral assay doses. According to Tierney et al. (2016), flavorants range from 1- to 20-fold the amount of nicotine in the traditional cigarette as well as ENDS, with an average flavor concentration of ∼12 mg/ml (Omaiye et al., 2019). It has been confirmed that 0.5 mg/kg nicotine is rewarding for mice in conditioned place preference (CPP) assays (Tapper et al., 2004; Henderson et al., 2016, 2017). Given this dose of nicotine, we determined the clinically relevant dose of farnesene is 0.5–10 mg/kg and used a dosing range of 0.1, 1.0, and 10 mg/kg for this study.

### Mice

All experiments were conducted in accordance with the guidelines for care and use of animals provided by the National Institutes of Health. Protocols were approved by the Institutional Animal Care and Use Committee at Marshall University. Mice were group housed on a standard 12/12 h light/dark cycle at 22°C and given food and water *ad libitum*. For all assays, we used α4-mCherryα6-GFP mice, originated from a C57BL/6J strain that are genetically modified to contain α4* and α6* nAChR fluorescent tags. These mice have been shown to exhibit similar levels of nAChRs to wild-type mice and behave similar to wild-type mice in CPP assays (Mackey et al., 2012; Srinivasan et al., 2016; Henderson et al., 2017; Avelar et al., 2019). Following CPP assays, mouse brains that were homozygous for α4-mCherry and transgenic for α6-GFP were used in confocal microscopy assays (20 mice; Fig. 3). For a portion of the behavioral experiments, wild-type littermate mice and The Jackson Laboratory C57BL/6J mice were used alongside α4-mCherryα6-GFP mice. For all experiments, we used adult (three to six months old) mice. Both male and female mice were used and numbers of each are detailed below in the methods for specific experiments and given in detail in corresponding figures.

Our genetically modified α4-mCherryα6-GFP mice were the result of crossing α4-mCherry homozygous knock-in mice (Srinivasan et al., 2016) with α6-GFP bacterial artificial chromosome (BAC) transgenic mice (Mackey et al., 2012). α4-mCherry knock-in mice are backcrossed to C57BL/6J mice every 10 generations while α6-GFP mice are continuously backcrossed to C57BL/6J mice (from The Jackson Laboratory; https://www.jax.org/strain/000664).

### Genotyping

On postnatal day 21, mice were weaned and housed with same-sex littermates. Concomitantly, tail biopsies were taken for genotyping analysis by PCR (Transnetyx). Only mice that were transgenic for α6-GFP and homozygous for α4-mCherry were used in confocal assays (see below), with the exception of α6-GFP and α4-mCherry mice used for normalized Förster resonance energy transfer (NFRET) controls.

### Locomotor assays

**Figure 1.**
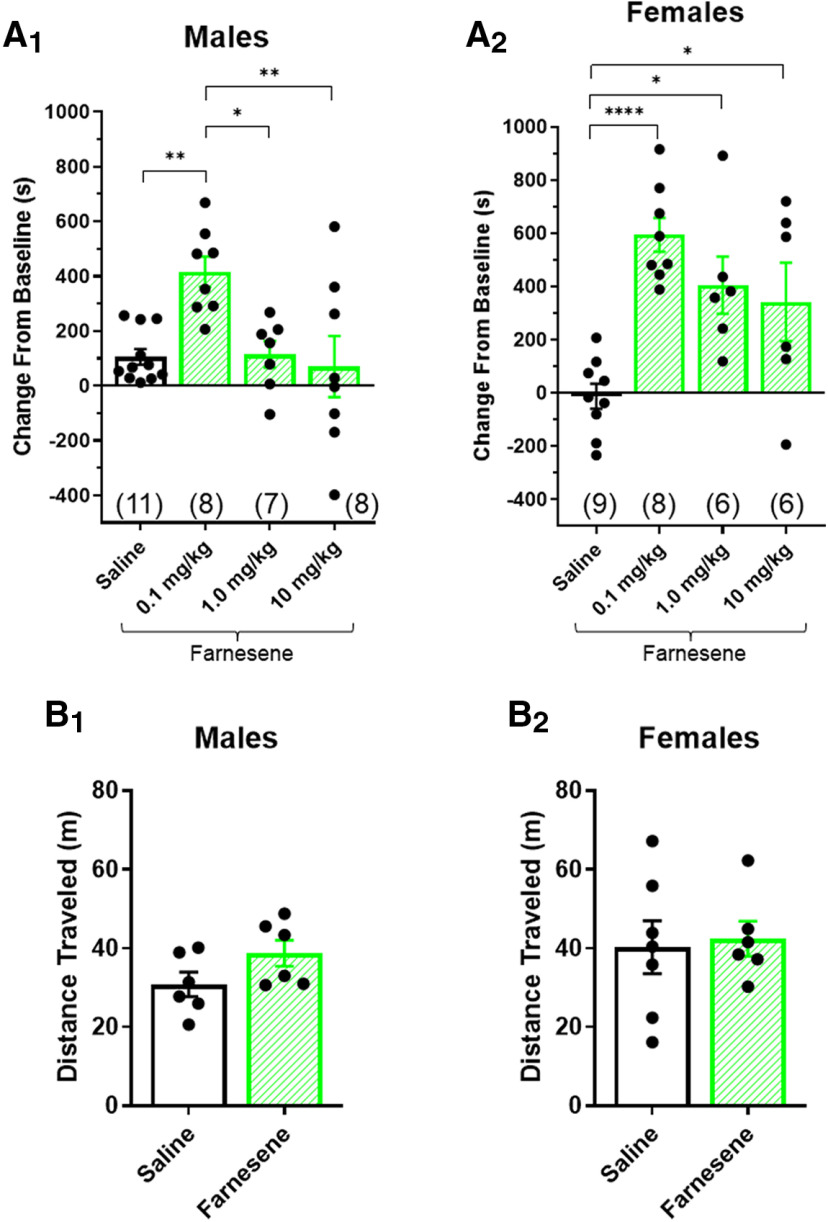
Farnesene-alone produces reward-related behavior in male and female mice. ***A_1,2_***, Male and female mice were administered saline or farnesene at doses of 0.1, 1.0, or 10 mg/kg in a CPP assay. ***B_1,2_***, Male and female mice were administered saline or 0.1 mg/kg farnesene in an open field locomotor assay. All data are mean ± SEM; *p <* *0.05, **p <* *0.01, ****p <* *0.0001; one-way ANOVA with *post hoc* Tukey (***A***) or unpaired *t* test (***B***). Exact *p* values are given in Results. Number of mice for each treatment group in CPP assays is indicated in parenthesis. Dots within bars represent the CPP scores or locomotor activity from individual mice within the designated treatment groups.

Adult male and female non-transgenic α4-mCherryα6-GFP mice (three to five months old) were habituated to the experimental room for ∼1 h before the experiment (*n* = 6–7 mice/sex). Mice were placed in an open field (40 × 40 × 36 cm) immediately after an intraperitoneal injection of saline or farnesene (0.1 mg/kg). Distance traveled over a period of 20 min was recorded using motion tracking software (SMART 3.0). Number of mice are listed in Figure 1*B*.

### CPP assays

**Figure 2.**
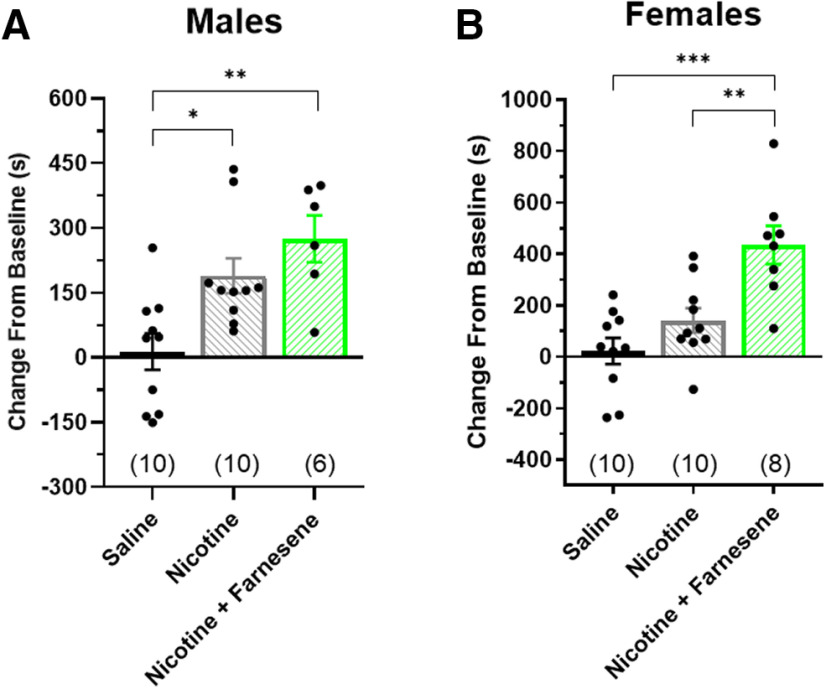
Farnesene (0.1 mg/kg) enhances nicotine reward-related behavior in both sexes. ***A***, ***B***, Male and female mice were administered saline, nicotine (0.5 mg/kg), or nicotine (0.5 mg/kg) plus farnesene (0.1 mg/kg) in a CPP assay. All data are mean ± SEM; *p <* *0.05, **p <* *0.01, ***p <* *0.005; one-way ANOVA with *post hoc* Tukey. Exact *p* values are given in Results. Number of mice for each treatment group is indicated in parenthesis, and dots within bars represent the CPP scores from individual mice within the designated treatment group.

CPP assays were conducted in a three-chamber spatial place-preference apparatus (Harvard Apparatus, PanLab) over a 10-d period, using male and female mice (Figures 1 and 2). Time spent in chambers was recorded by motion tracking software (SMART 3.0). The test consisted of three stages: pre-test, injections, post-test. An unbiased protocol was used where “drugs” (saline, nicotine (0.5 mg/kg), farnesene alone (0.1, 1.0, or 10 mg/kg), or 0.1 mg/kg farnesene plus 0.5 mg/kg nicotine) were given immediately before confinement in the right white/gray chamber on drug days and saline was given immediately before confinement in the left white/black chamber on saline days. Each conditioning period lasted 20 min. Before the use of experimental mice during each stage, a scrap mouse of the same sex was used to deposit odors for 20 min. In the pre-test stage, mice were placed in the central chamber and given 20 min of free access to all chambers. Drug-naive mice that spent >65% of their time in one of the two conditioning chambers were removed from the study and the remaining mice were counter-balanced, similar to previously published methods (Neugebauer et al., 2011; Einstein et al., 2013; Lee et al., 2020). Eight males and five females were excluded. During stage 2, intraperitoneal injections were given in the white/gray chamber (saline control, farnesene, nicotine, or farnesene plus nicotine, dissolved in saline) or white/black chamber (saline). The mice received their designated drug injections on days 2, 4, 6, and 8, and received saline injections on days 3, 5, 7, and 9. In the post-test stage, mice were again placed in the central chamber and given 20 min of free access to all chambers. Adult male and female mice, three to six months old, were used in CPP assays (60 males and 57 females total). Previous reports have shown that the use of α4-mCherryα6-GFP mice have displayed no differences in nicotine reward-related behavior as tested by CPP in comparison to C57BL/6J mice (Henderson et al., 2017; Avelar et al., 2019). Sex differences are further discussed in Results. Data are expressed as a change in baseline preference between the post-test and pre-test. Drug treatments were blind to experimenters until all data analysis was completed.

### Confocal imaging of mouse brain slices

α4-mCherryα6-GFP mice used in CPP assays were also used in microscopy assays. Following the completion of CPP assays, mice were euthanized with CO_2_ and subjected to a swift cardiac perfusion with 10-ml ice-cold saline to reduce autofluorescence in the mCherry emission range. Brains were then swiftly removed, flash frozen with acetone and dry ice, and then stored at −80°C. Brains were coronally sectioned (20 μm) using a cryostat, mounted with Vectashield (Vector labs, H-1000), and coverslipped. We targeted bregma −3.1 mm (anterior-posterior limits of −2.9 to −3.3 mm) because this region gave the most consistent sections that contained a large portion of the VTA, substantia nigra pars reticulata (SNr), and substantia nigra pars compacta (SNc) in a single slice.

A Leica SP5 TCSII confocal microscope was used to excite α6-GFP and α4-mCherry at 488 and 561 nm, respectively; 20× images with a 10× digital zoom were collected for the quantitative measurements of α4-mCherry and α6-GFP neuron raw integrated density (RID). NFRET was calculated using the PixFRET ImageJ plug-in to identify α4α6* nAChRs.

**Figure 3.**
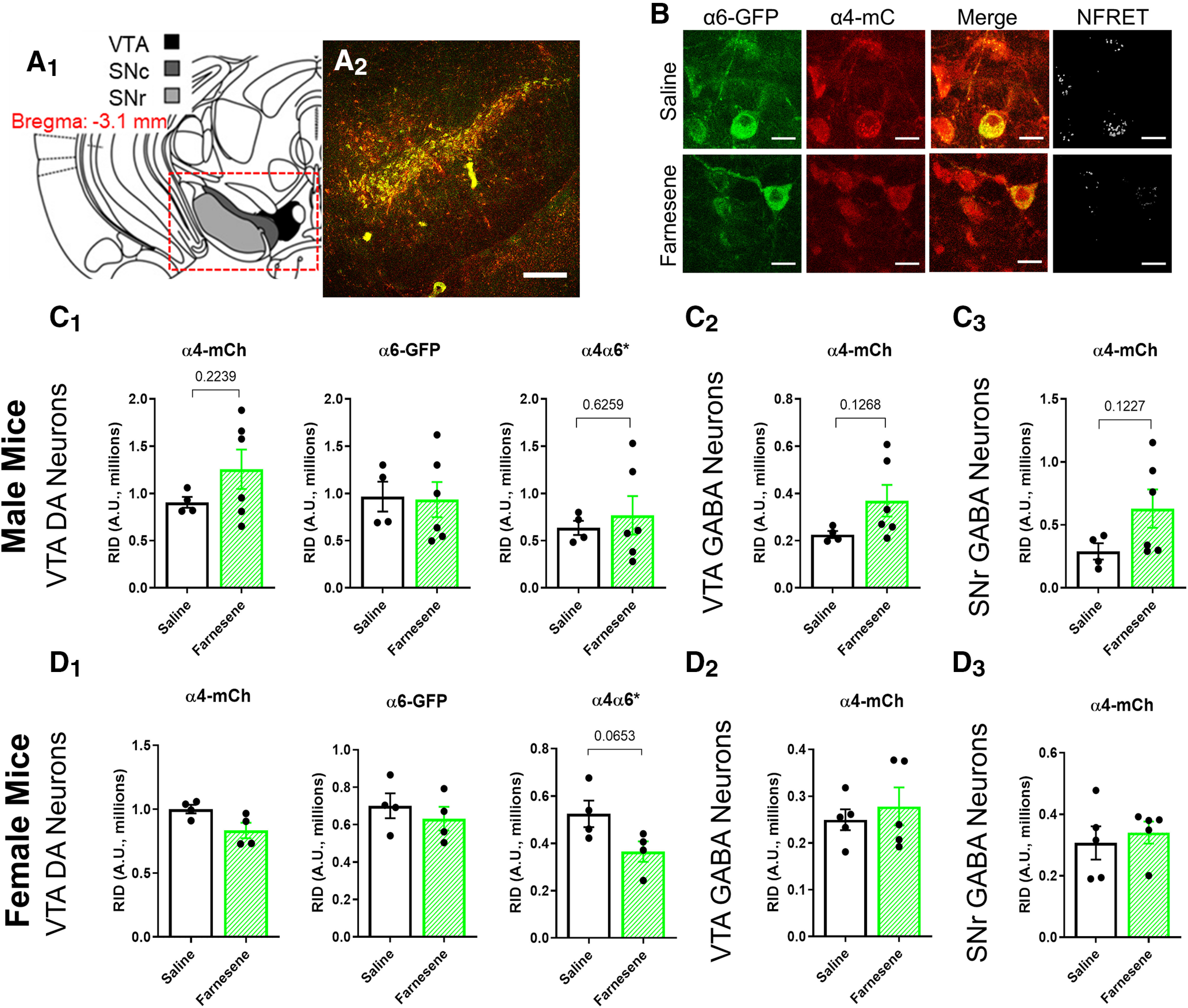
Farnesene treatment has no effect on nAChR number in the midbrain. ***A_1_***, Schematic of target mouse brain region (bregma −3.1 mm). ***A_2_***, Sample 10× image of a mouse coronal brain section at approximately bregma −3.1 mm. Scale bar, 250 μm. ***B***, Sample images of saline and farnesene treated VTA dopamine neurons. Scale bar, 15 μm. ***C***, ***D***, RID of α4*, α6*, and α4α6* nAChRs of VTA dopamine neurons (***C_1_***, ***D_1_***), α4* nAChRs of VTA GABA neurons (***C_2_***, ***D_2_***), and α4* nAChRs of SNr GABA neurons (***C_3_***, ***D_3_***) in saline-treated and farnesene-treated (0.1 mg/kg) male (***C***) and female (***D***) mice. All data are mean ± SEM. Unpaired *t* test. Dots indicate the RID values from individual mice.

All experimenters were blind to drug treatment until all data analysis was completed. Approximately 30–60 VTA dopamine neurons and ≥30 VTA and SNr GABA neurons were imaged. Data from these images were averaged to provide RID values for each mouse. A total of 20 mice were used in confocal assays, aged three to five months (*n* provided in Fig. 3).

### Neuro-2a cell culture and transient transfections

Mouse neuroblastoma 2a (neuro-2a) cells were cultured in the following medium: MEM with 5% fetal bovine serum, 100 IU/ml penicillin, and 100 μg/ml streptomycin. Cells were plated by adding 90,000 or 50,000 cells (microscopy and electrophysiology, respectively) to poly-D-lysine-coated 35-mm glass-bottom imaging dishes (MatTek Corporation) and cultured in a humidified incubator (37°C, 95% air, 5% CO_2_). Cells were transfected with 500 ng of each nAChR subunit cDNA plasmid (α4-mCherry, α4-GFP, and β2wt or α4-mCherry, α6-GFP, and β2wt depending on intended subtype target and FRET pairing). Following plating procedures, plasmid DNA was mixed with 250 μl of Opti-MEM and Lipofectamine-3000 was separately added to the Opti-MEM. After 5 min at 24°C, the two solutions were combined and incubated at 24°C for 25 min. Plated neuro-2a cells then received the mixed solution and were incubated for 24 h. The next day, Opti-MEM was removed, and the cells received growth medium. 500 nm filter-sterilized farnesene (or sham treatment) was added after replacing the Opti-MEM with standard culture medium. Twenty-four hours after drug/sham addition, neuro-2a cells were imaged on a confocal microscope (discussed above) or examined using electrophysiological methods (discussed below).

### Whole-cell patch-clamp electrophysiology

Using brain slices from three- to five-month-old male and female α6-GFP mice, we identified putative dopamine (pDA) neurons of the VTA for recordings. Dopamine neurons of the lateral VTA selectively express α6* nAChRs making our α6-GFP mice suitable for identifying pDA neurons in electrophysiological assays. After recent work detailing the presence of α6* nAChRs on medial VTA glutamate neurons (Yan et al., 2018), we restricted our recordings to the lateral VTA to increase our chance of accurately identifying pDA neurons. Following behavioral assays, mice were anesthetized with CO_2_, and then cardiac perfusion was performed using ice-cold NMDG-based artificial CSF (NMDG-ACSF) saturated with 95% O_2_/5% CO_2_ (carbogen) containing the following: 93 mm NMDG, 2.5 mm KCl, 1.2 mm NaH_2_PO_4_, 10 mm MgSO_4_, 0.4 mm CaCl_2_, 30 mm NaHCO_3_, 5 mm Na-ascorbate, 3 mm Na-pyruvate, 2 mm thiourea, and 25 mm glucose. Brains were removed and kept in agarose for slicing with a Compresstome VF-300-OZ (Precisionary Instruments). Coronal brain sections (250 μm) were cut into cold carbogenated NMDG-ACSF to obtain slices containing the VTA (target bregma −3.1 mm; anterior-posterior limits of −2.7 to −3.5 mm) and were then allowed to recover at 32°C in carbogenated NMDG-ACSF for 12–15 min. Following this, slices were transferred to standard ACSF containing the following: 125 mm NaCl, 2.5 mm KCl, 1.2 mm NaH_2_PO_4_, 1.2 mm MgCl_2_, 2.4 mm CaCl_2_, 26 mm NaHCO_3_, and 11 mm glucose for 1 h at 32°C. One hour later, slices were transferred to the recording chamber and continuously perfused with carbogenated ACSF (1.5–2.0 ml/min) at 32°C.

Neurons were visualized with an upright microscope (Axio Examiner A1, Zeiss) equipped with an Axiocam 702 mono using DIC near infrared illumination. Blue illumination was used to visualize α6-GFP presence in pDA neurons. Whole-cell patch-clamp techniques were used to record electrophysiological signals with an Integrated Patch-Clamp Amplifier (Sutter). Patch electrodes had resistances of 4–10 MΩ when filled with intrapipette solution: 135 mm K gluconate, 5 mm KCl, 5 mm EGTA, 0.5 mm CaCl_2_, 10 mm HEPES, 2 mm Mg-ATP, and 0.1 mm GTP. Recordings were sampled at ≥10 kHz. The junction potential between patch pipette and bath solutions was nulled just before gigaseal formation. Series resistance was monitored without compensation throughout experiments using SutterPatch software. The recording sessions for neurons were terminated if the series resistance changed by >20%. Nicotine and farnesene (dissolved in ACSF at pH 7.4) applications were applied using pressure microinjection (Picospritzer III, Parker) at 5 psi. Drug concentrations and duration of applications are given in Results. While microinjections of nicotine mitigate many of the complications of usage in brain slices (diffusion in and out of tissue and cells), we used a maximum of three nicotine puffs per brain slice.

For the recordings of spontaneous EPSCs (sEPSCs), bath perfusion of ACSF was switched to an ACSF solution containing 100 μm picrotoxin (Sigma-Aldrich, catalog number 124-87-8) to block GABA_A_ receptors. After 5 min, pDA neurons in the VTA were voltage clamped at −65 mV to record sEPSCs.

For cultured cells, 50,000 neuro-2a cells were plated onto sterilized 12-mm circular glass coverslips, placed in 35-mm culture dishes and cultured in a humidified incubator (37°C, 95% air, 5% CO_2_). Cells were transfected as described above. For patching of neuro-2a cells, we used extracellular solution containing the following: 140 mm NaCl, 5 mm KCl, 2 mm CaCl_2_, 1 mm MgCl_2,_ 10 mm HEPES, and 10 mm glucose (320 mOsm, pH set to 7.3 with Tris-base). For voltage-clamp experiments, neuro-2a cells were voltage clamped at a holding potential of −55 mV. To avoid nAChR desensitization by repetitive nicotine application, we applied drug puffs at ∼3-min intervals and continually perfused the recording chamber with extracellular solution.

### Statistical analysis

**Figure 4.**
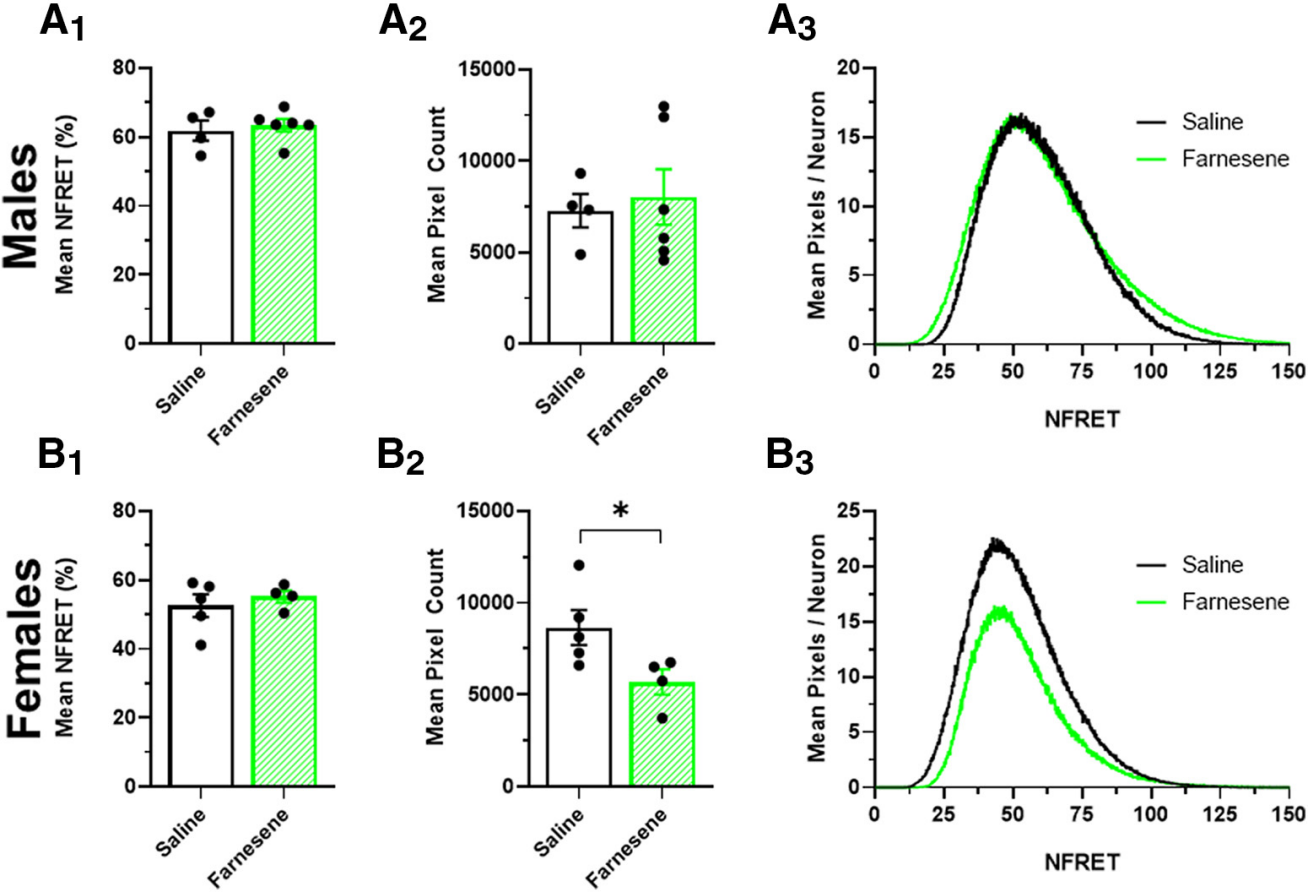
Farnesene alters the stoichiometry of α4α6β2* nAChRs in VTA DA neurons. Mean NFRET percentage (***A_1_***, ***B_1_***,), mean NFRET pixel count (***A_2_***, ***B_2_***), and mean pixels/neuron histograms (***A_3_***, ***B_3_***) for saline-treated and farnesene-treated (0.1 mg/kg) VTA dopamine neurons in male (***A***) and female (***B***) mice. All data are mean ± SEM; *p <* *0.05; unpaired *t* test. Exact *p* values are given in Results. Dots within bars represent the values from individual mice within the designated treatment group; *n* > 40 neurons per mouse per treatment group.

**Figure 5.**
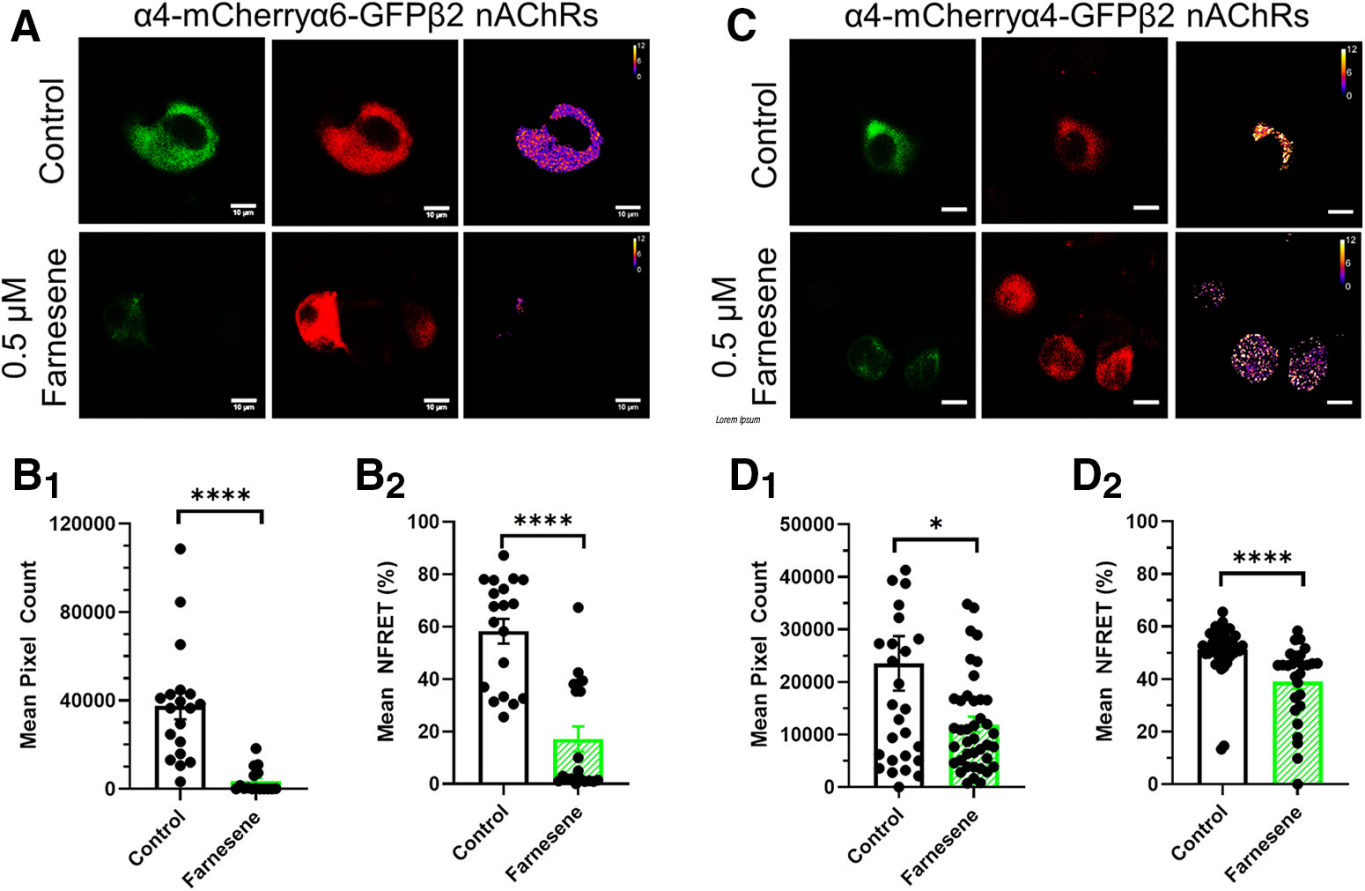
Farnesene favors high-sensitivity nAChRs in neuro-2a cells. Representative neuro-2a cells transfected with α4-mCherry, α4-GFP or α6-GFP, and β2wt nAChR subunits to produce (***A***) α4-mCherryα6-GFPβ2 nAChRs or (***C***) α4-mCherryα4-GFPβ2 nAChRs. Scale bar, 10 μm. Mean NFRET pixel count (***B_1_***, ***D_1_***) and NFRET percentage (***B_2_***, ***D_2_***) treated as control or with 0.5 μm farnesene for (***A***) α4-mCherryα6-GFPβ2 nAChRs or (***C***) α4-mCherryα4-GFPβ2 nAChRs. All data are mean ± SEM; *p <* *0.05, ****p <* *0.001; unpaired *t* test. Exact *p* values are given in Results. Dots within bars represent the values from individual cells within the designated treatment group; *n* > 30 cells per condition.

**Figure 6.**
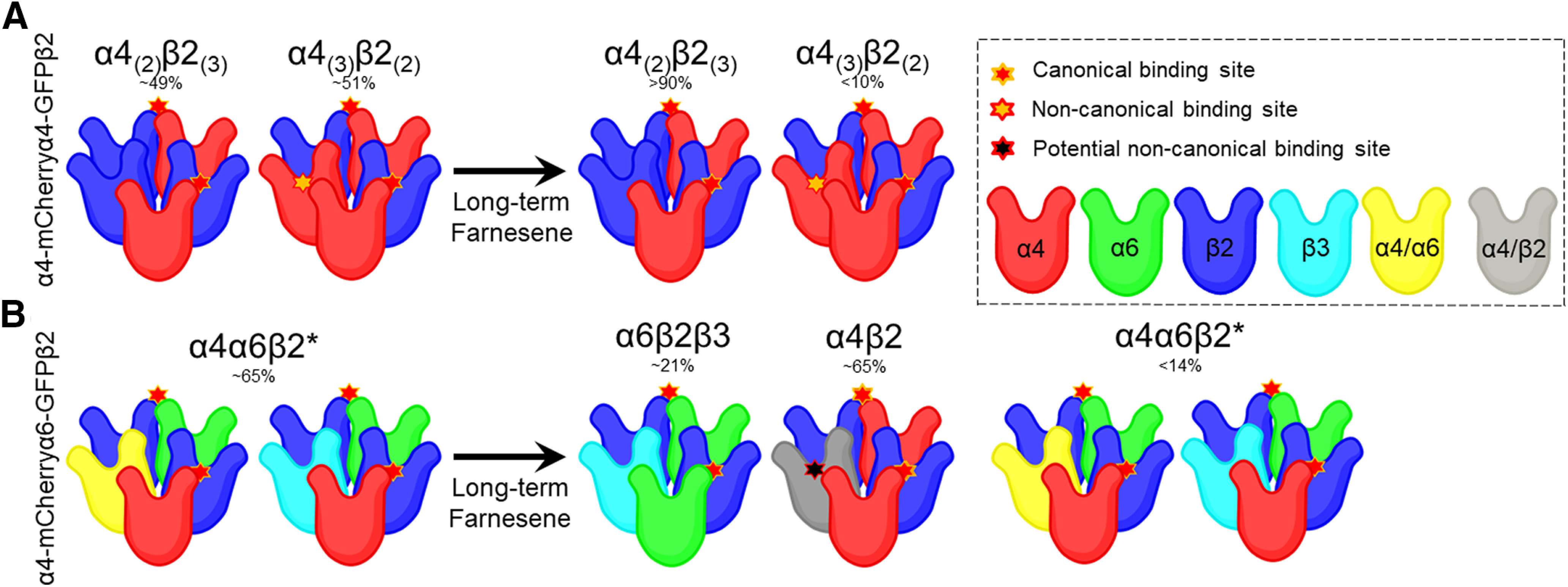
Farnesene favors high-sensitivity nAChRs in neuro-2a cells. ***A***, α4β2 nAChRs assemble in two stoichiometries, and we observed that farnesene treatment shifts a mixed population of HS and LS α4β2 nAChRs to a majority of HS α4β2 nAChRs. ***B***, In examining α4α6β2 nAChRs, under control treatments, ∼65% of the population are α4α6β2 nAChRs while the remainder is likely α4β2 nAChRs. Following treatment with farnesene, <14% of the nAChRs are α4α6β2 nAChRs.

**Figure 7.**
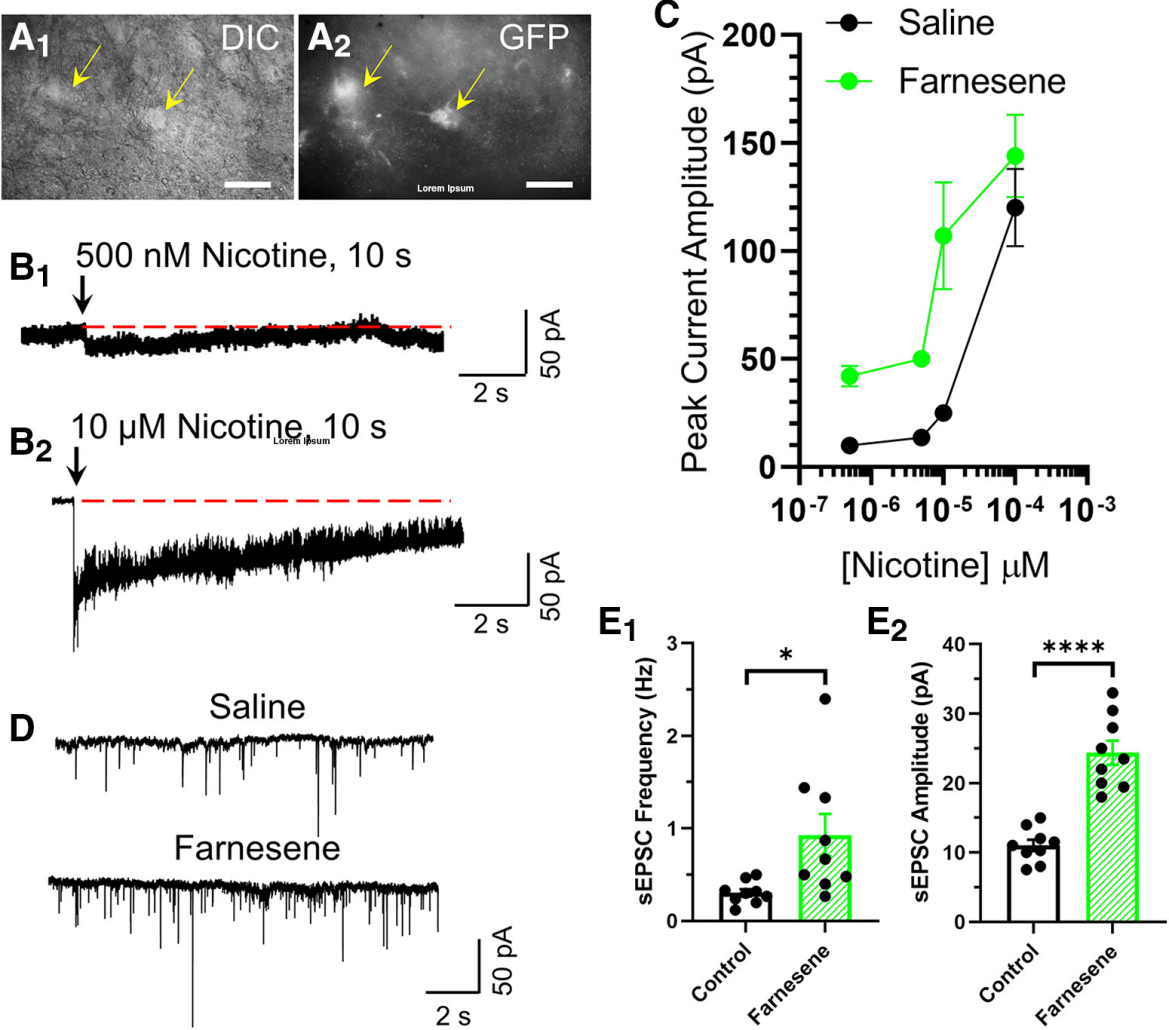
Farnesene enhances the affinity and potency of nicotine. Representative images of VTA pDA neurons in a brain slice (bregma −3.1) were identified through the presence of α6-GFP nAChRs in IR-DIC (***A_1_***) and GFP fluorescence (***A_2_***) imaging modes. Scale bars, 20 μm. ***B***, Representative inward currents from VTA pDA neurons (α6-GFP-positive) with 10-s applications of 500 nm (***B_1_***) or 10 μm (***B_2_***) nicotine in voltage-clamp mode. Arrows indicate start of nicotine puff application and dotted red lines indicate baseline before puff and the duration of nicotine application. ***C***, Average nicotine concentration response of peak-current amplitude of VTA pDA neurons (*n* = 7 neurons/4 mice and 5 neurons/3 mice per nicotine concentration for saline-treated and farnesene-treated mice, respectively). ***D***, Representative waveforms of sEPSCs from VTA pDA neurons recorded from saline-treated or farnesene-treated mice in the presence of 30 μm picrotoxin. ***E***, Mean sEPSC frequency (***E_1_***) and amplitude (***E_2_***) in saline-treated and farnesene-treated mouse brain slices (*n* = 9 neurons/4 mice and 9 neurons/3 mice for saline-treated and farnesene-treated mice, respectively). For all assays, drug treatments were consistent with the CPP assay paradigm using 0.1 mg/kg farnesene. ***C***, ***E_I,2_***, Data are mean ± SEM *p <* *0.05, ****p <* *0.0001; unpaired *t* test. Exact *p* values are given in Results. Dots within bars represent the values from individual neurons within the designated treatment group.

**Figure 8.**
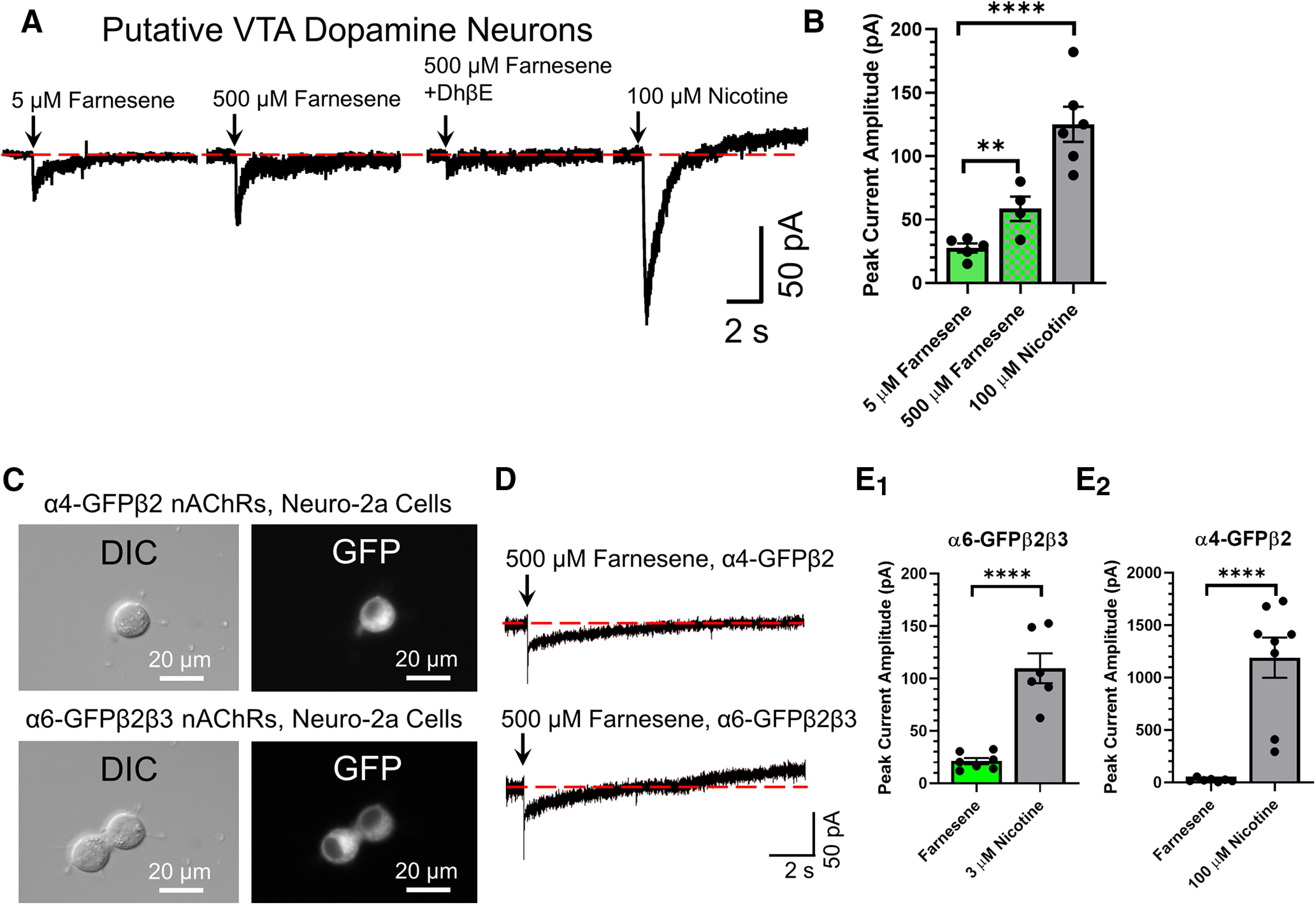
Farnesene acts as a partial agonist on nAChRs. ***A***, ***B***, Voltage-clamp recordings from putative VTA dopamine neurons. ***A***, Five and 500 μm farnesene and 100 μm nicotine were applied to putative VTA dopamine neurons. The β2* nAChR antagonist, DhβE (0.3 μm) blocked inward currents stimulated by 500 μm farnesene. ***B***, Mean peak current amplitude for farnesene and nicotine applications on pDA neurons in the VTA. ***C***–***E***, Voltage-clamp recordings from neuro-2a cells transiently transfected to contain α4-GFPβ2 and α6-GFPβ2β3 nAChRs. ***C***, Representative images of neuro-2a cells that contain α4-GFPβ2 or α6-GFPβ2β3 nAChRs. ***D***, Representative inward currents stimulated by 300-ms applications of 500 μm farnesene on neuro-2a cells containing α4-GFPβ2 or α6-GFPβ2β3 nAChRs. ***E_1,2_***, Mean peak current amplitude of 500 μm farnesene and nicotine applications (3 and 100 μm nicotine for α6-GFPβ2β3 and α4-GFPβ2 nAChRs, respectively) on neuro-2a cells containing nAChRs. ***B***, ***E_1,2_***, Data are mean ± SEM; **p <* *0.01, ****p < 0.0001; one-way ANOVA with *post hoc* Tukey (***B***) or unpaired *t* test (***E***). Dots represent data from individual neurons or cells. Exact *p* values are given in Results.

All results are presented as mean ± SEM and all statistical analyses were performed using GraphPad Prism. CPP data (Figs. 1*A*, 2) were analyzed with either a one-way (drug factor) or two-way ANOVA (sex × drug × interaction) with a *post hoc* Tukey for means comparison. Unpaired data (Figs. 1*B*, 3, 4, 5, 6, 7, 8*E*_1,2_) were analyzed by *t* test. Figure 8*B* was analyzed with a one-way ANOVA (drug factor) with a *post hoc* Tukey for means comparison. Complete statistical tests and results are displayed in the statistical table provided (Table 1). Power analyses (G*Power software; www.gpower.hhu.de) were used to determine efficient sample sizes (Tables 2–Tables 6).

**Table 1:** Statistical tests and results

Figure	Type of test	Interaction/main effect	Statistical data
1, 2	Two-way ANOVA	Sex × drug	*F*_(5,88)_ = 3.045, *p* = 0.0140
1, 2	Two-way ANOVA	Sex	*F*_(1,88)_ = 10.55, *p* = 0.0016
1, 2	Two-way ANOVA	Drug	*F*_(5,88)_ = 12.21, *p* < 0.0001
1*A_1_*	One-way ANOVA		*F*_(3,30)_ = 5.98, *p* = 0.0025
1*A_1_*	*Post hoc* Tukey		Saline vs 0.1 mg/kg; *p* = 0.0065
1*A_1_*	*Post hoc* Tukey		Saline vs 1.0 mg/kg; *p* = 0.9997
1*A_1_*	*Post hoc* Tukey		Saline vs 10 mg/kg; *p* = 0.9770
1*A_1_*	*Post hoc* Tukey		0.1 vs 1.0 mg/kg; *p* = 0.0203
1*A_1_*	*Post hoc* Tukey		0.1 vs 10 mg/kg; *p* = 0.0047
1*A_1_*	*Post hoc* Tukey		1.0 vs 10 mg/kg; *p* = 0.9680
1*A_2_*	One-way ANOVA		*F*_(3,25)_ = 9.81, *p* = 0.0002
1*A_2_*	*Post hoc* Tukey		Saline vs 0.1 mg/kg; *p* < 0.0001
1*A_2_*	*Post hoc* Tukey		Saline vs 1.0 mg/kg; *p* = 0.0126
1*A_2_*	*Post hoc* Tukey		Saline vs 10 mg/kg; *p* = 0.0402
1*A_2_*	*Post hoc* Tukey		0.1 vs 1.0 mg/kg; *p* = 0.4631
1*A_2_*	*Post hoc* Tukey		0.1 vs 10 mg/kg; *p* = 0.2245
1*A_2_*	*Post hoc* Tukey		1.0 vs 10 mg/kg; *p* = 0.9669
1*B_1_*	Unpaired *t* test		Saline vs farnesene (males), *p* = 0.111
1*B_2_*	Unpaired *t* test		Saline vs farnesene (females), *p* = 0.801
2*A*	One-way ANOVA		*F*_(2,23)_ = 8.506, *p* = 0.0017
2*A*	*Post hoc* Tukey		Saline vs nicotine; *p* = 0.0173
2*A*	*Post hoc* Tukey		Saline vs nicotine + farnesene; *p* = 0.0022
2*A*	*Post hoc* Tukey		Nicotine vs nicotine + farnesene; *p* = 0.4280
2*B*	One-way ANOVA		*F*_(2,25)_ = 13.04, *p* = 0.0001
2*B*	*Post hoc* Tukey		Saline vs nicotine; *p* = 0.2916
2*B*	*Post hoc* Tukey		Saline vs nicotine + farnesene; *p* = 0.0001
2*B*	*Post hoc* Tukey		Nicotine vs nicotine + farnesene; *p* = 0.0041
3*C_1_*	Unpaired *t* test		[α4, *p* = 0.2239], [α6, *p* = 0.9065], [α4α6, *p* = 0.6259]
3*C_2_*	Unpaired *t* test		*p* = 0.1268
3*C_3_*	Unpaired *t* test		*p* = 0.1227
3*D_1_*	Unpaired *t* test		[α4, *p* = 0.0515], [α6, *p* = 0.4842], [α4α6, *p* = 0.0653]
3*D_2_*	Unpaired *t* test		*p* = 0.5572
3*D_3_*	Unpaired *t* test		*p* = 0.6156
4*A_1_*	Unpaired *t* test		*p* = 0.6343
4*A_2_*	Unpaired *t* test		*p* = 0.7225
4*B_1_*	Unpaired *t* test		*p* = 0.5285
4*B_2_*	Unpaired *t* test		*p* = 0.0480
5*B_1_*	Unpaired *t* test		*p* < 0.0001
5*B_2_*	Unpaired *t* test		*p* < 0.0001
5*D_1_*	Unpaired *t* test		*p* = 0.0149
5*D_2_*	Unpaired *t* test		*p* < 0.0001
7*E_1_*	Unpaired *t* test		*p* = 0.0002
7*E_2_*	Unpaired *t* test		*p* < 0.0001
8*B*	One-way ANOVA		*F*_(2,12)_ = 23.05, *p* < 0.0001
8*B*	*Post hoc* Tukey		5 μm farnesene vs 500 μm farnesene; *p* = 0.1823
8*B*	*Post hoc* Tukey		5 μm farnesene vs 100 μm nicotine; *p* < 0.0001
8*B*	*Post hoc* Tukey		500 μm farnesene vs 100 μm nicotine, *p* = 0.0031
8*E_1_*	Unpaired *t* test		*p* < 0.0001
8*E_2_*	Unpaired *t* test		*p* < 0.0001

**Table 2:** G*Power statistics, CPP (**Figs. 1, 2)**

Input parameters		Output parameters	
Test family	*F* tests	Noncentrality parameter	16.94
Test type	ANOVA: one-way	Critical F	2.21
Type of analysis	A priori	Numerator df	7
Effect size	0.55	Denominator df	48
α err prob	0.05	Total sample size	56
Power	0.8	Sample size/group	7
Number of groups	8	Actual Power	0.811

Sample size indicates number of mice needed per treatment group.

**Table 3:** G*Power statistics, locomotor behavior (Fig. 1*B_1,2_* **)**

Input parameters		Output parameters	
Test family	*t* tests (two tails)	Noncentrality parameter	3.21
Test type	Biserial model	Critical *t*	2.23
Type of analysis	A priori	df	10
Effect size	0.68	Total sample size	12
α err prob	0.05	Sample size/group	6
Power	0.8	Actual power	0.825
Number of groups	2		

Sample size indicates the number of neurons/cells needed for sufficient power.

**Table 4:** G*Power statistics, fluorescence microscopy (**Figs. 3, 4)**

Input parameters		Output parameters	
Test family	*t* tests (two tails)	Noncentrality parameter	3.53
Test type	Biserial model	Critical *t*	2.57
Type of analysis	A priori	df	5
Effect size	0.8	Total sample size	7
α err prob	0.05	Sample size/group	≥3
Power	0.8	Actual Power	0.803
Number of groups	2		

Sample size indicates number of mice needed per treatment group.

**Table 5:** G*Power statistics, brain slice electrophysiology (sEPSCs; **Fig. 7)**

Input parameters		Output parameters	
Test family	*t* tests (two tails)	Noncentrality parameter	3.25
Test type	Biserial model	Critical *t*	2.26
Type of analysis	A priori	df	9
Effect size	0.7	Total sample size	11
α err prob	0.05	Sample size/group	6–7
Power	0.8	Actual Power	0.823
Number of groups	2		

Sample size indicates the number of neurons needed for sufficient power.

**Table 6:** G*Power statistics, electrophysiology (inward currents; **Fig. 8)**

Input parameters		Output parameters	
Test family	*t* tests (two tails)	Noncentrality parameter	3.40
Test type	Biserial model	Critical *t*	2.36
Type of analysis	A priori	df	7
Effect size	0.75	Total sample size	9
α err prob	0.05	Sample size/group	≥5
Power	0.8	Actual Power	0.830
Number of groups	2		

Sample size indicates the number of neurons/cells needed for sufficient power.

## Results

### Farnesene-alone prompts reward-related behavior

Here, we examine a chemical flavorant of green apple, farnesene, for its ability to alter reward-related behavior. Using male and female α4-mCherryα6-GFP mice (C57BL/6J background) and farnesene doses of 0.1, 1.0, and 10 mg/kg, we conducted CPP assays to measure reward-related behavior. The previous report that examined another green apple flavorant, farnesol, observed a sex-dependent effect on mice in CPP assays (Avelar et al., 2019). Accordingly, we hypothesized that farnesene may produce a sex-dependent effect given the fact that its chemical structure is very similar to farnesol. We observed a sex-dependent and drug-dependent effect in our CPP assays with the above farnesene doses, as well as a significant interaction between sex and drug factors (two-way ANOVA (sex × drug dose × interaction); sex factor: *F*_(1,88)_ = 10.55, *p *=* *0.002; drug factor: *F*_(5,88)_ = 12.21, *p *<* *0.0001; and interaction factor: *F*_(5,88)_ = 3.045, *p *= 0.014). Following the significant sex-dependent effect, we examined the effect of farnesene on reward-related behavior separately for male and female mice.

We noted a significant effect of farnesene in male and female mice [males: one-way ANOVA, *F*_(3,30)_ = 5.98, *p *=* *0.003 (Fig. 1*A_1_*); females: one-way ANOVA, *F*_(3,25)_ = 9.81, *p *=* *0.0002 (Fig. 1*A_2_*)]. In males, farnesene at a dose of 0.1 mg/kg prompted a significant rewarding effect compared with saline-treated (*p *=* *0.0065), 1.0 mg/kg farnesene-treated (*p *=* *0.020), and 10 mg/kg farnesene-treated (*p *=* *0.005) mice. Higher doses of farnesene in male mice did not exhibit a significant change from baseline compared with saline, which may mimic nicotine’s inverted-U dose response in CPP assays. On the other hand, female mice exhibited a significant change from baseline with all farnesene doses when compared with saline (1.0 mg/kg, *p *=* *0.013; 10 mg/kg, *p *=* *0.040; Fig. 1*A_2_*), with the greatest change from baseline at 0.1 mg/kg (*p *<* *0.0001; Fig. 1*A_2_*). We note, farnesene doses tested may need to be higher in order for the females to observe an inverted-U response similar to the males. Similarly, we may need to test lower doses in both males and females to determine the threshold dose for reward-related behavior. Overall, these data support the idea that farnesene at 0.1 mg/kg is rewarding on its own in both male and female mice. Following these observations, follow-up assays were performed with 0.1 mg/kg dosing in male and female mice.

Additionally, we conducted locomotor assays to determine the effects of acute farnesene treatment on locomotor behavior in male and female mice. It is well understood that nicotine increases locomotor activity in mice (Wall et al., 2017), and was recently determined that green apple flavorant, farnesol, also increased this behavior (Avelar et al., 2019). Here, male and female mice were administered intraperitoneal injections of saline or 0.1 mg/kg farnesene, and locomotor activity was assessed in an open field test (Fig. 1*B_1,2_*). Unlike the previous reports, farnesene did not significantly alter the ambulatory behavior of either male or female mice compared with saline treatment [males, *p *<* *0.111 (Fig. 1*B_1_*); females, *p *<* *0.801 (Fig. 1*B_2_*)].

### Farnesene enhances nicotine reward

Because of the fact that many ENDS users prefer flavored nicotine-containing products, and the findings that farnesene-alone is rewarding (Fig. 1*A*), it is important we study the combining effect of farnesene and nicotine on reward-related behavior. To do so, we performed additional CPP assays using saline, nicotine, and nicotine combined with the peak farnesene dose (0.1 mg/kg). Many previous reports have used 0.5 mg/kg nicotine (intraperitoneal) to produce a rewarding effect in C57 mice (Tapper et al., 2004). Accordingly, we selected this dose for these CPP assays. Only male mice exhibited significant nicotine reward-related behavior (one-way ANOVA, *p *=* *0.017 and *p *=* *0.292 for males and females, respectively; Fig. 2*A*,*B*); however, in both sexes, we noted a significant nicotine + farnesene effect compared with saline [one-way ANOVA; males, *p *=* *0.002 (Fig. 2*A*); females, *p *=* *0.0001 (Fig. 2*B*)]. Additionally, both sexes exhibited enhanced rewarding effects in the nicotine + farnesene group compared with nicotine alone, but only females exhibited a significant enhancement (*p *=* *0.004).

### Despite effects on reward-related behavior, farnesene does not upregulate nAChRs on VTA dopamine neurons

Following behavioral assays, we performed confocal microscopy to observe potential changes in nAChR levels based on the long-standing knowledge that chronic nicotine exposure upregulates nAChRs (Nashmi et al., 2003, 2007; Henderson et al., 2014; Srinivasan et al., 2016) and that green apple flavorant, farnesol, worked in a similar manner (Avelar et al., 2019). Following the well-characterized effects of nAChR upregulation in midbrain neurons, we focused on dopamine and GABA neurons in the VTA and GABAergic neurons in the SNr (Fig. 3*A*). Using α4-mCherryα6-GFP mice, we examined α4β2* (* denotes other subunits may be present), α6β2*, and α4α6β2* nAChR density of pDA and GABA neurons in the VTA and SNr in response to saline or farnesene treatment. Here, we used the same mice that were employed in CPP assays to maintain dosing relevant to reward-related behavior. To aid in the identification of pDA neurons in the VTA, we used α6-GFP fluorescence as a marker, as α6-GFP nAChR subunits are highly expressed in dopamine neurons in the lateral VTA (Mackey et al., 2012; described further in methods for electrophysiology). Given that GFP and mCherry are FRET pairings, we used pixel-based FRET methods to identify nAChRs that contain both α4 and α6 nAChR subunits (Fig. 3*B*). Changes in nAChR number was determined by quantifying a change in RID of α4-mCherry or α6-GFP fluorescence. Unlike the previous study by Avelar et al. (2019), demonstrating farnesol’s ability to upregulate nAChRs (2019), farnesene treatment produced no significant changes in nAChR number within pDA or GABA neurons in the VTA or SNr (Fig. 3*C*,*D*).

### Despite the absence of upregulation, farnesene alters stoichiometry of nAChRs

In addition to examining the upregulation of nAChRs in mouse brain slices, we also investigated farnesene-induced changes in nAChR stoichiometry. Nicotine has long been known to selectively upregulate high-sensitivity nAChRs, including α4* and α6* nAChRs (Kuryatov et al., 2005; Srinivasan et al., 2011), while menthol (in the absence of nicotine) and cytisine have been shown to upregulate low-sensitivity nAChRs (Srinivasan et al., 2012; Henderson et al., 2016). α4β2 nAChRs exist in two stoichiometries: high sensitivity (α4_(2)_β2_(3)_) and low sensitivity (α4_(3)_β2_(2)_; Nelson et al., 2003). α6* nAChR stoichiometries are characterized as α6β2, α6β2β3, or α4α6β2β3 (Lindstrom et al., 1987; Xiao et al., 2011; Henderson et al., 2014).

To determine farnesene’s effects on nAChR stoichiometry, we used a pixel-based, NFRET method (Srinivasan et al., 2012). Aside from quantifying the RID of α4*, α6*, and α4α6* nAChRs, we additionally examined the effect of farnesene treatment on NFRET intensity and pixel count as both are informative measurements regarding the change in number of nAChR pentamers that contain both an α4 and α6 nAChR subunit (Fig. 4). In both males and females, we saw no significant changes in the mean NFRET intensity between saline and farnesene treatment groups (Fig. 4*A_1_*,*B_1_*). Yet, in females-only, we observed a significant decrease in the mean pixel count following farnesene treatment (*p *=* *0.048; Fig. 4*B* 2,3). This indicates that farnesene reduces the number of α4α6* nAChRs on VTA dopamine neurons in female mice. Given that females demonstrated the greatest range in farnesene-induced reward-related behavior, this finding may explain why we only see an effect on pixel count in female mice.

Given that our mouse model only allows analysis of changes in α4α6* nAChR stoichiometry, we used *in vitro* methods to examine both α4α6β2 and α4β2 nAChR stoichiometry following farnesene exposure. Accordingly, we studied nAChR stoichiometry changes using neuro-2a cells that were transiently transfected with α4-mCherry, α6-GFP, and β2wt or α4-mCherry, α4-GFP, and β2wt nAChR subunits to examine changes in α4α6β2 and α4β2 nAChR stoichiometry (Fig. 5*A*,*C*, respectively). Cells transiently transfected to contain α4-mCherryα6-GFPβ2 nAChRs (Fig. 5*A*) were treated with control media or 0.5 μm farnesene for 24 h. We selected 0.5 μm farnesene to match previous studies with the similarly structured terpene, menthol (Henderson et al., 2016) and farnesol (Avelar et al., 2019). In farnesene-treated cells, we noted a significant decrease in the mean pixel count (*p < *0.0001; Fig. 5*B_1_*) and the mean NFRET percentage (*p < *0.0001; Fig. 5*B_2_*), which indicates a significant decrease in the number of α4-α6 nAChR subunit pairings. Thus, the data obtained with *in vitro* methods matches those obtained using mouse brain slices. Cells transiently transfected to contain α4-mCherryα4-GFPβ2 nAChRs (Fig. 5*C*) were also treated with control media or 0.5 μm farnesene, and we also observed a significant decrease in mean pixel count (*p = *0.0149; Fig. 5*D_1_*) and mean NFRET percentage (*p *<* *0.0001; Fig. 5*D_2_*). Accordingly, this indicates a decrease in the number of low-sensitivity α4_(3)_β2_(2)_ nAChRs. These results are further summarized in Figure 6. Here, we show that long-term farnesene treatment promotes the inclusion of high-sensitivity nAChRs by transitioning the number of low-sensitivity α4_(3)_β2_(2)_ (Fig. 6*A*) and α4α6β2 (Fig. 6*B*) nAChRs to a majority of high-sensitivity α4_(2)_β2_(3)_ nAChRs.

### Farnesene alters VTA dopamine neuron function

Although we found no significant changes in nAChR upregulation or downregulation on VTA neurons, the changes in stoichiometry observed in VTA dopamine neurons and neuro-2a cells suggest farnesene alters stoichiometry, potentially toward high-sensitivity α4β2 nAChRs. To examine this in an *in vivo* model, we used brain slice whole-cell electrophysiology. Given that high-sensitivity and low-sensitivity α4β2* nAChRs can be functionally distinguished through concentration-response relationships (Nelson et al., 2003; Xiao et al., 2009), we used similar methods to determine whether farnesene treatment altered nAChR stoichiometry on putative VTA dopamine neurons. Mackey et al. (2012) have previously demonstrated α6 nAChRs exhibit complete overlap with tyrosine hydroxylase in VTA dopamine neurons. Thus, several previous investigations have used α6-GFP fluorescence, in addition to other electrophysiological markers, as a method to identify dopamine neurons within the VTA and SNc (Mackey et al., 2012; Henderson et al., 2016, 2017; Avelar et al., 2019). However, more recent investigations have shown that α6-GFP nAChRs are present on glutamate neurons in the medial portions of the VTA (Yan et al., 2018). To increase our chances of identifying pDA neurons, we targeted α6-GFP positive cells (Fig. 7*A_1,2_*) in the lateral VTA that exhibited classical electrophysiological markers of dopamine neurons (Margolis et al., 2006, 2008): hyperpolarizing sag (*I*_h_), firing frequency, and action potential widths >2 ms. Together, these features have been proven to provide a robust method of identifying pDA neurons (Mackey et al., 2012; Henderson et al., 2017). We used coronal brain slices obtained from saline or 0.1 mg/kg farnesene treated mice using a dosing paradigm that matches the above CPP protocol and targeted bregma −3.1 to match confocal microscopy assays. Putative VTA dopamine neurons were voltage-clamped at −65 mV and 0.5, 5, 10, and 100 μm concentrations of nicotine were applied using localized pressure injection with a micropipette (5 psi, 10-s applications; Fig. 7*B_1,2_*,*C*). In the farnesene-treated condition, we noted a leftward shift in the concentration response of nicotine, indicative of a shift toward more high-sensitivity nAChRs (Fig. 7*C*). In addition to recording nicotine-stimulated inward currents, we also recorded sEPSCs in pDA neurons (Fig. 7*D*). Here, we observed that chronic farnesene increased the baseline frequency (*p *=* *0.0163) and amplitude (*p *<* *0.0001) of sEPSCs on putative VTA dopamine neurons compared with saline treatment (Fig. 7*E_1,2_*).

### Farnesene acts as a partial agonist of nAChRs

The previous report on farnesol, another green apple flavorant, showed that farnesol did not stimulate nAChR-mediated currents on its own but likely functions as a noncompetitive antagonist (Avelar et al., 2019). To examine farnesene’s pharmacological actions, we first started with acute applications on putative VTA dopamine neurons (Fig. 8*A*,*B*). We observed that 5 and 500 μm farnesene stimulates inward currents in putative VTA dopamine neurons (mean peak current amplitudes of 27.5 ± 3.3 and 58.5 ± 9.6 pA, respectively; Fig. 8*B*). We note that 0.5 μm farnesene (consistent with *in vitro* NFRET assays) did not produce any notable inward currents (data not shown). Using ACSF containing DhβE (0.3 μm), we observed that farnesene-induced (500 μm) inward currents are dependent on β2* nAChRs (Fig. 8*A*). Finally, we used 100 μm nicotine to obtain a comparative efficacy for farnesene and determined that farnesene possessed ≤44.0 ± 7.3% efficacy compared with nicotine (at 100 μm) on putative VTA dopamine neurons. Five and 500 μm farnesene had a significantly lower peak current amplitude compared with 100 μm nicotine (*p* < 0.0001 and *p* < 0.0031, respectively). In examining peak current amplitudes of nAChRs on dopamine neurons, it is difficult to isolate exactly which subtypes are activated. To address this, we used neuro-2a cells transiently transfected to contain either α4-GFPβ2 or α6-GFPβ2β3 nAChRs (Fig. 8*C*). These two nAChR subtypes exhibit distinct sensitivities to nicotine and can be maximally stimulated by 3 μm nicotine (α6-GFPβ2β3 nAChRs) or 100–300 μm nicotine (α4-GFPβ2 nAChRs; Henderson et al., 2014). For neuro-2a cells containing nAChRs, we applied 500 μm farnesene (300-ms applications) and observed mean peak current amplitudes of 21.2 ± 3.0 and 23.8 ± 6.1 pA for α6-GFPβ2β3 and α4-GFPβ2 nAChRs, respectively (Fig. 8*E_1,2_*). Using 3 and 100 μm nicotine, we observed mean peak current amplitudes of 109.7 ± 14.3 pA (*p *<* *0.0001) and 1190.4 ± 192.3 pA (*p *<* *0.0001) for α6-GFPβ2β3 and α4-GFPβ2 nAChRs, respectively (Fig. 8*E_1,2_*). Thus, the comparative efficacy of farnesene to nicotine is 19.3 ± 2.7% and 2.1 ± 0.6% for α6-GFPβ2β3 and α4-GFPβ2 nAChRs, respectively.

## Discussion

With the growing popularity of ENDS products among all ages and the large preference for flavored e-liquids (Schneller et al., 2018; Huang et al., 2019; Mead et al., 2019), the goal of our study was to determine how one green apple flavorant, farnesene, may affect vaping-related behaviors using behavioral, neurobiological, and neurophysiological assays. Menthol has long been the most studied flavorant for its large prevalence in combustible cigarettes and is known to enhance nicotine reward (Henderson et al., 2016, 2017) and nicotine reinforcement (Wang et al., 2014; Biswas et al., 2016). Yet, with the increase in flavorant production with >15,000 ENDS flavorant options available to the vaping community (Hsu et al., 2018), there is an urgent need to understand how flavorants alter neurobiology and neurophysiology. A recent investigation revealed farnesol (a green apple flavorant similar in structure to farnesene) behaves similarly to menthol by enhancing nicotine reward but differs in the fact that it causes reward-related behavior on its own (Henderson et al., 2017; Avelar et al., 2019). Here, we noted similar findings: farnesene, a green apple flavorant, causes reward-related behavior on its own. However, one key difference from the previous report is that farnesene produces reward in male and female mice while farnesol only produced an effect with males (Avelar et al., 2019). Additionally, farnesol caused significant upregulation of α6-containing nAChRs (Avelar et al., 2019), but farnesene did not upregulate nAChRs. Instead, farnesene was observed to alter nAChR stoichiometry to promote the expression of more high-sensitivity nAChRs as evidenced by electrophysiology and NFRET assays in mouse brain slices and cultured cells.

This is a very significant finding considering that >90% of adolescents ENDS users and >70% of adult ENDS users prefer flavored products (Schneller et al., 2018; Mead et al., 2019). Given the absence of restrictions on most flavored ENDS, it is important we document the effects that flavorants have on altering the addictive nature of nicotine and determining potential addictive properties in flavorants alone. Although we observed reward-related behavior with farnesene in both sexes, we did note sex differences in our CPP assays. First, females exhibited reward-related behavior at all doses of farnesene examined (0.1, 1, and 10 mg/kg) while males exhibited reward to only 0.1 mg/kg farnesene. This may be because of the fact that females metabolize farnesene faster than males, similar to nicotine (Matta et al., 2007). Additionally, females did not exhibit significant nicotine reward with 0.5 mg/kg nicotine. To date, nicotine dose-response data have primarily been in male mice, and those in females are only given via subcutaneous injection (Kota et al., 2008). Because of the lack of nicotine dose–response curve data using intraperitoneal injections in females, this may explain the lack of significant nicotine reward at the given dose. Although there is a significant change from baseline for nicotine + farnesene in both males and females, the change from baseline is less than farnesene-alone in both sexes. While others have reported significant CPP with 0.5 mg/kg nicotine in CPP assays using both sexes, this could highlight the need to re-examine nicotine dose responses with both sexes in this assay. Additionally, we would like to note that acute farnesene did not significantly alter locomotor behavior compared with saline treatment in male or female mice. Although nicotine and farnesol were observed to increase locomotor activity in mice, previous studies have demonstrated these changes are likely mediated through α6β2* and α4α6β2* nAChRs (Drenan et al., 2010; Cohen et al., 2012). Given that the farnesene-induced behavioral effects shown here are likely mediated through high-sensitivity α4β2 nAChRs, this may be the reason we did not see any significant changes in locomotor behavior between farnesene-treated or saline-treated mice.

Although farnesol and farnesene exhibit similar behavioral results, they differ in the cellular aspects that drive reward-related behavior. Based on previously published findings that chronic nicotine exposure promotes the upregulation of high-sensitivity nAChRs on VTA dopamine, VTA/SNr GABA, and habenular neurons and the recent evidence that farnesol acted in a similar manner, our initial investigations focused on the number of α4*, α6*, and α4α6* nAChRs present on dopamine and GABA neurons as a consequence of farnesene exposure. We noted no significant changes between farnesene and saline treatment groups. The reward-related behaviors we noted may not be attributed to nAChR upregulation but, instead, by changes in nAChR stoichiometry. While many are well informed regarding the various subtypes of nAChRs, there also exist distinct stoichiometries that confer different sensitivities to nicotine (Nelson et al., 2003; Tapia et al., 2007). Both our microscopy measurements (NFRET) and brain slice electrophysiology data support the fact that farnesene treatment, consistent with CPP dosing, produces more high-sensitivity nAChRs on VTA dopamine neurons. This increase in high-sensitivity (likely α4β2) nAChRs results in not only increased sensitivity to nicotine, but also to the endogenous neurotransmitter ACh.

In the case of changes in stoichiometry, we note there are limitations to our methods. Currently, we possess only the capability to measure α4α6* nAChR stoichiometry *in vivo*. Thus, we required the use of heterologous cells and transfection methods in an *in vitro* system to conduct fluorescence microscopy assays to study changes in α4β2 nAChR stoichiometry. We acknowledge that this may not truly represent *in vivo* systems and we note a distinct difference between the *in vivo* and *in vitro* findings with α4α6* nAChRs (compare Figs. 4, 5*B_1,2_*). These two systems also differ in the time dependency of nAChR upregulation and stoichiometry changes. Nicotine or flavorant induced nAChR upregulation and changes in stoichiometry occur over the course of 10–12 d *in vivo* (Henderson et al., 2016, 2017; Nashmi et al., 2007), whereas the *in vitro* system findings occur in 24 h (Srinivasan et al., 2011). This difference in time dependency for *in vitro* preparations is accompanied by the lacking complexity of a cell’s environment *in vivo* (Fu et al., 2019). Stoichiometry changes within *in vivo* systems are not only brain region specific (Fu et al., 2019), but they also rely on unique cellular machinery that is absent in our *in vitro* model. Despite this, our transfection methods follow validated protocols that have matched several *in vivo* mouse and human studies (Srinivasan et al., 2011; Henderson et al., 2017), and we are able to detect a change in nAChR stoichiometry that is consistent with our findings using brain slice electrophysiology as both suggest the presence of more high-affinity nAChRs.

Farnesene treatment significantly increased the frequency and amplitude of sEPSCs. The excitatory inputs to the VTA dopamine neurons we studied are local and distal glutamate neurons (medial VTA, prefrontal cortex) and distal cholinergic neurons (peduncular pontine tegmentum and laterodorsal tegmentum). The inhibitory inputs are GABA neurons in the VTA, rostromedial tegmentum, and interpeduncular nucleus. Thus, the changes in sEPSC frequency and amplitude are likely because of changes in glutamatergic or GABAergic local inputs given that we used a coronal brain slice preparation. While many of the cholinergic inputs are distal and are not present in a coronal slice preparation, some cholinergic signaling is maintained, and this is likely a source of elevated sEPSC frequency and amplitude. Regardless, the enhanced frequency and amplitude of sEPSCs in VTA dopamine neurons suggests that farnesene triggers enhanced activity that may drive dopamine release through the mesolimbic pathway and thus contribute to reward-related behavior.

In addition to this, we observed that farnesene itself acts as a partial agonist, likely through α6-containing nAChRs, as it exhibited very low efficacy on α4β2 nAChRs transiently transfected into neuro-2a cells. Here, we must acknowledge that a dose of 500 μm is likely not vaping relevant, but it was necessary to determine the comparable efficacy to nicotine. Despite this, 5 μm farnesene still produced detectable inward currents in VTA dopamine neurons, and this suggests that farnesene may also weakly stimulate nAChRs in the VTA. Here, further investigations would need to be conducted to determine the human vaping-relevant concentrations of farnesene that would be present in the brain and how this specific concentration alters neurophysiology. Even with these caveats, this finding is significant, especially in the rising popularity of zero-nicotine e-liquids. Green apple flavors (depending on the specific flavorants present) may directly stimulate nAChRs, and this could be the rationale for their popularity.

The data reported in this study suggest that (1) farnesene enhances nicotine reward and displays reward-related behavior through the increase in high-sensitivity nAChRs on VTA dopaminergic neurons; (2) that farnesene changes nAChR stoichiometry and results in an enhanced affinity and potency of nicotine and ACh; and (3) that this ultimately leads to enhanced excitatory postsynaptic currents on VTA dopamine neurons and thus greater likelihood of action potential generation. Overall, this may explain why flavored ENDS are growing in popularity and may contribute to low cessation rates. The finding of enhanced nicotine reward when flavorants are present and the fact that flavorants-alone may be rewarding, further indicates the importance of studying flavorants for their potential to alter vaping-related behaviors. However, there are components of this local VTA circuitry that still need to be elucidated: (1) how are VTA glutamatergic inputs and their nAChRs altered by farnesene; (2) how are α4β2 nAChRs on VTA GABA neurons altered functionally; and (3) what is the net effect of farnesene on dopamine release. Additionally, there are several green apple flavorants that are still unknowns in the scope of how they alter vaping-related behavior (methylbutyl acetate, hexyl acetate, and ethyl acetate). Based on these findings, it is of importance we continue to investigate ENDS flavors for their role in nicotine addiction. With the continuous rise in ENDS use, especially among the adolescent population, it is critical we depict that ENDS flavors are not a simple additive to the devices, instead they are an enhancer to the addictive properties of nicotine and a potential threat to zero-nicotine flavored ENDS users as well.

## Synthesis

Reviewing Editor: Christie Fowler, University of California, Irvine Decisions are customarily a result of the Reviewing Editor and the peer reviewers coming together and discussing their recommendations until a consensus is reached. When revisions are invited, a fact-based synthesis statement explaining their decision and outlining what is needed to prepare a revision will be listed below. The following reviewer(s) agreed to reveal their identity: Cassandra Gipson.

Both of the reviewers were positive about the manuscript and thought the data presented were of high interest. However, several significant concerns were raised in the reviews and consultation session, including the lack of full statistical reporting, consistency in data reporting on the graphs (individual data points should be included), and conclusion statements that were not supported by the findings or need further discussion. An important control condition was identified as missing - whether the transgenic mice show altered nicotine- and farnesene-induced conditioned place preference compared to wild type mice. Further, both reviewers agreed that Figure 9 should be removed. Specific reviewer comments to address follow below:

Reviewer 1

The manuscript titled “Green apple e-cigarette flavorant farnesene triggers reward-related behavior by promoting high-sensitivity nAChRs in the ventral tegmental area” is an interesting combination of studies showing that farnesene, a chemical in the green apple flavorant of ENDS products, can induce conditioned place preference on its own, and enhance nicotine-induced conditioned place preference in both male and female mice. Further, this manuscript shows that prolonged exposure to farnesene alters the stoichiometry of nicotinic acetylcholine receptors (nAChRs) in the ventral tegmental area (VTA), and alters dopaminergic physiology within this key node of the mesolimbic reward pathway. The studies demonstrate compelling evidence that this chemical alters the neurobiology and behavior associated with nicotine use. However, issues outlined below have decreased enthusiasm for the manuscript, and should be addressed.

1. An important missing component of these studies is whether the transgenic mice show altered nicotine- and farnesene-induced conditioned place preference compared to wild type mice. Further, do these mice have altered expression of the nAChR subunit that is genetically targeted and modified to fluoresce?

2. It is unclear why a scrap mouse was used to deposit an odor of the same sex prior to experimental animal placement in the chambers. Why was this necessary? Were boxes cleaned between animals appropriately? Were there male- and female-specific chambers assigned to prevent odors between sexes from impacting outcomes?

3. Authors claim that animals were excluded from these studies that displayed strong (>65%) preference for one side of the CPP chamber. This seems inappropriate, as counterconditioning against a strong preference would be a stronger argument that this flavorant can induce reward-relevant behavior.

4. The statistical analyses are confusing. The methods section has a statistical analysis section, but it does not explain the methodology for what analyses were conducted when, nor does it explain what variables are for each analysis. For example, the authors utilize a two-way ANOVA but do not state the variables within the first paragraph of the results section. Further, the authors report the results of a two-way ANOVA after describing sex-dependent effects on CPP, but this does not convey any information that would allow the reader to understand what the ANOVA actually showed. Was there a sex x side preference interaction? Is this reported F and p value reflective of that? What did the rest of the ANOVA show? If the authors did not get a significant interaction with sex, the subsequent one-way ANOVAs with each sex are inappropriate unless there were a priori hypotheses to justify running independent ANOVAs on each sex.

5. The authors report that G Power was used for power analyses, but the results of these power analyses are not reported. How were power analyses conducted on neurophysiological experiments? Was by animal or by cell used? These details are necessary to report.

6. Does farnesene alter locomotor activity during the conditioning phase? This is an important outcome as it is a manipulation check to ensure that the drug is having physiological effects. Further, it is another outcome that can be compared to nicotine.

7. The authors expose animals to farnesene and report conditioned place preference results. In a separate experiment, the authors examine altered stoichiometry of nAChRs following farnesene exposure. The question here is whether the exposure to farnesene in vitro reflects exposure due to volitional intake in vivo, and if changes in nAChR stoichiometry are leading to these changes in VTA dopamine physiology. The authors attempt to address this in the discussion, but it should be expanded. Limitations should also be included regarding what the current data do and do not support.

8. In Figure 7, there are 7 neurons/condition. How many animals is reflected in each condition?

9. Figure 9 does not seem completely necessary, and does not add to the manuscript given that it appears to contain assumptions rather than be reflective of current data (e.g., neurons in orange appear to signal acetylcholine, but this was not examined here). Further, in the discussion, Figure 9 is discussed in terms don’t seem to be reflected in the data presented (e.g., “This increase in high-sensitivity... nAChRs results in not only increased sensitivity to nicotine, but also to the endogenous neurotransmitter acetylcholine”. Unless it was missed, it doesn’t seem that this was directly measured here and thus seems inappropriate.

10. Generally, the manuscript needs some attention. Details are outlined below:

a. In the abstract, the authors state that they “observed farnesene-induced inward currents of ventral tegmental area dopamine neurons” but do not state compared to what, and do not identify what this change means (how is this neurobiological change relevant to nicotine addiction/ENDS use?)

b. In the introduction, the last paragraph describes what was done in the studies. Each study, however, is separated by a semicolon, which is odd sentence structure.

c. In the results section, the first paragraph contains numerous sentences that belong in the introduction and are inappropriate in the results section.

d. Minor: Page 10, “this data supports” should be “these data support”.

Reviewer 2

n this manuscript, the authors present the results of a series of experiments to suggest that repeated exposure to farnesene facilitates molecular and behavioral changes that facilitate reward-related behaviors in both male and female mice. Farnesene represents a common flavor additive to e-cigarette solutions, and the authors provide substantial evidence that it facilitates changes in neuronal activity in the VTA to drive these changes in behavior.

In general, the manuscript is well-written and the figures (and legends) are clear. The experiments appear to be well-designed and the results are appropriately interpreted in the context of prior research and limitations. The authors use multiple convergent techniques (behavior, imaging, electrophysiology) to form their conclusions. Overall, findings have important potential implications for the potential addiction-facilitating properties of the e-cigarette flavor additive farnesene. Given the quality of the work, my comments are relatively minor.

(1) The authors describe their criteria for identifying putative dopamine neurons (lines 307-309) as a robust method for identifying putative dopamine neurons. We suggest that the authors include a reference here supporting this method of cell-type identification, or provide additional details if this is a novel approach to identifying putative dopamine neurons.

(2) The authors may want to consider identifying another representative trace from the saline group with a more stable baseline (Figure 7D).

(3) The authors chose to use represent individual data points in Figure 5 and Figure 7, but did include them in all additional figures containing data. The authors should revise their figures to have consistent inclusion or exclusion of individual data points.

(4) Figure 9 provides a summary of the findings that includes speculation regarding changes in nAChR sensitivity in GABAergic neurons in the VTA. Since this manuscript did not contain inhibitory recordings (or direct imagine evidence) supporting nAChR changes in the VTA, some of the conclusions illustrated in Figure 9 remain somewhat speculative. The authors should consider revising this figure to reflect the need for future work to elucidate the role of farnesene on inhibitory signaling in the VTA.

(5) Discussion of the implications of translation of these findings to human use (lines 232-233) are somewhat speculative as the current experiments are done in mice. This would be best suited for the Discussion section at the end of the manuscript.

## Author Response

Reviewer 1

The manuscript titled “Green apple e-cigarette flavorant farnesene triggers reward-related behavior by promoting high-sensitivity nAChRs in the ventral tegmental area” is an interesting combination of studies showing that farnesene, a chemical in the green apple flavorant of ENDS products, can induce conditioned place preference on its own, and enhance nicotine-induced conditioned place preference in both male and female mice. Further, this manuscript shows that prolonged exposure to farnesene alters the stoichiometry of nicotinic acetylcholine receptors (nAChRs) in the ventral tegmental area (VTA), and alters dopaminergic physiology within this key node of the mesolimbic reward pathway. The studies demonstrate compelling evidence that this chemical alters the neurobiology and behavior associated with nicotine use. However, issues outlined below have decreased enthusiasm for the manuscript, and should be addressed.

1. An important missing component of these studies is whether the transgenic mice show altered nicotine- and farnesene-induced conditioned place preference compared to wild type mice. Further, do these mice have altered expression of the nAChR subunit that is genetically targeted and modified to fluoresce?

Response: Previous work has shown that mice containing these α4-mCherry and α6-GFP nAChR mutations do not behave differently than C57BL/6J mice (in conditioned place preference assays) and contain similar levels of nAChR protein. We have added the appropriate references to refer to this previous work.

2. It is unclear why a scrap mouse was used to deposit an odor of the same sex prior to experimental animal placement in the chambers. Why was this necessary? Were boxes cleaned between animals appropriately? Were there male- and female-specific chambers assigned to prevent odors between sexes from impacting outcomes?

Response: CPP assays were initially conducted with the use of a scrap mouse at the direction of a more-senior scientist whom has conducted CPP with mice using nicotine for an extensive period of time (the person is not mentioned due to the double-blind review). Reviewers for this work and another have both raised a concern for the use of a scrap mouse and state it is unnecessary if boxes are cleaned properly. Thus, we have independently compared the use and absence of a scrap mouse and have noted no significant difference in CPP with nicotine or saline in C57 or our genetically modified mice. However, in order to remain consistent, we continued our CPP assays with scrap mice. We do clean the chambers in between individual mice as is necessary to prevent odors from impacting outcomes.

3. Authors claim that animals were excluded from these studies that displayed strong (>65%) preference for one side of the CPP chamber. This seems inappropriate, as counterconditioning against a strong preference would be a stronger argument that this flavorant can induce reward-relevant behavior.

Response: Other labs use similar exclusion criteria for mice after the pre-test in unbiased CPP assays (Lee et al., 2020; Neugebauer et al., 2011; Einstein et al., 2013, referenced in revised manuscript). In reviewing our video recordings, the mice that exhibit >65% preference for one chamber are most likely due to a fear or anxiety-induced behavior that results in the mouse ‘freezing’ in one chamber. Although we aim to limit anxiety and fear to the best of our ability, we cannot eliminate this behavior in some of our mice.

4. The statistical analyses are confusing. The methods section has a statistical analysis section, but it does not explain the methodology for what analyses were conducted when, nor does it explain what variables are for each analysis. For example, the authors utilize a two-way ANOVA but do not state the variables within the first paragraph of the results section. Further, the authors report the results of a two-way ANOVA after describing sex-dependent effects on CPP, but this does not convey any information that would allow the reader to understand what the ANOVA actually showed. Was there a sex x side preference interaction? Is this reported F and p value reflective of that? What did the rest of the ANOVA show? If the authors did not get a significant interaction with sex, the subsequent one-way ANOVAs with each sex are inappropriate unless there were a priori hypotheses to justify running independent ANOVAs on each sex.

Response: We have updated the Statistical Analysis section in our methods to include more detail regarding the variables tested and for which data sets they were performed on. Additionally, we have updated the Results section to include more details. We did observe a significant interaction with sex using a two-way ANOVA. All F and p values are provided in the revised manuscript in the first paragraph of the results. The significant interaction with sex justifies the subsequent one-way ANOVAs with each sex.

5. The authors report that G Power was used for power analyses, but the results of these power analyses are not reported. How were power analyses conducted on neurophysiological experiments? Was by animal or by cell used? These details are necessary to report.

Response: The inputs and outputs of our G*Power analysis are now reported in Tables 2 - 5. For the neurophysiology, the calculations were based upon neurons/cells (with triplicate recordings). We typically obtain suitable recordings from two neurons per mouse. To account for the expected number of mice we need for electrophysiology we take the results of our power analysis, divide by 2, to determine the number of mice necessary.

6. Does farnesene alter locomotor activity during the conditioning phase? This is an important outcome as it is a manipulation check to ensure that the drug is having physiological effects. Further, it is another outcome that can be compared to nicotine.

Response: In an open field test, farnesene did not alter locomotor activity. We have added this data to the revised version of our manuscript

7. The authors expose animals to farnesene and report conditioned place preference results. In a separate experiment, the authors examine altered stoichiometry of nAChRs following farnesene exposure. The question here is whether the exposure to farnesene in vitro reflects exposure due to volitional intake in vivo, and if changes in nAChR stoichiometry are leading to these changes in VTA dopamine physiology. The authors attempt to address this in the discussion, but it should be expanded. Limitations should also be included regarding what the current data do and do not support.

Response: This is an excellent point and we failed to address this important distinction in the previous version of our manuscript. The use of transfected cells does present as many limitations as it does benefits when considering translational impact. We have expanded our discussion to further parse out what our in vitro findings mean and what their limitations are. Despite these limitations, we do emphasize the in vitro findings do match our electrophysiological findings using brain slice preparations.

8. In Figure 7, there are 7 neurons/condition. How many animals is reflected in each condition?

Response: We have revised the legend for Figure 7 to include the number of mice used to collect the brain slice electrophysiology data. For the concentration response of nicotine, we used 4 and 3 mice for control and farnesene-treated mice, respectively. For the experiments including sEPSCs we used 4 and 3 mice for control and farnesene-treated mice, respectively.

9. Figure 9 does not seem completely necessary, and does not add to the manuscript given that it appears to contain assumptions rather than be reflective of current data (e.g., neurons in orange appear to signal acetylcholine, but this was not examined here). Further, in the discussion, Figure 9 is discussed in terms don’t seem to be reflected in the data presented (e.g., “This increase in high-sensitivity... nAChRs results in not only increased sensitivity to nicotine, but also to the endogenous neurotransmitter acetylcholine”. Unless it was missed, it doesn’t seem that this was directly measured here and thus seems inappropriate.

Response: We have since removed figure 9 as it was a strong assumption based on the data shown here.

10. Generally, the manuscript needs some attention. Details are outlined below:

a. In the abstract, the authors state that they “observed farnesene-induced inward currents of ventral tegmental area dopamine neurons” but do not state compared to what, and do not identify what this change means (how is this neurobiological change relevant to nicotine addiction/ENDS use?)

b. In the introduction, the last paragraph describes what was done in the studies. Each study, however, is separated by a semicolon, which is odd sentence structure.

c. In the results section, the first paragraph contains numerous sentences that belong in the introduction and are inappropriate in the results section.

d. Minor: Page 10, “this data supports” should be “these data support”.

Response: We have addressed each of these points by further expanded on point ‘a’ in the abstract and made the adjustments noted in points ‘b-d.’

Reviewer 2

n this manuscript, the authors present the results of a series of experiments to suggest that repeated exposure to farnesene facilitates molecular and behavioral changes that facilitate reward-related behaviors in both male and female mice. Farnesene represents a common flavor additive to e-cigarette solutions, and the authors provide substantial evidence that it facilitates changes in neuronal activity in the VTA to drive these changes in behavior.

In general, the manuscript is well-written and the figures (and legends) are clear. The experiments appear to be well-designed and the results are appropriately interpreted in the context of prior research and limitations. The authors use multiple convergent techniques (behavior, imaging, electrophysiology) to form their conclusions. Overall, findings have important potential implications for the potential addiction-facilitating properties of the e-cigarette flavor additive farnesene. Given the quality of the work, my comments are relatively minor.

(1) The authors describe their criteria for identifying putative dopamine neurons (lines 307-309) as a robust method for identifying putative dopamine neurons. We suggest that the authors include a reference here supporting this method of cell-type identification, or provide additional details if this is a novel approach to identifying putative dopamine neurons.

Response: We have provided citations of previously published reports that have used a combination of α6-GFP fluorescence, firing frequency, action potential width, and presence of Ih, to identify putative dopamine neurons.

(2) The authors may want to consider identifying another representative trace from the saline group with a more stable baseline (Figure 7D).

Response: We have revised Figure 7 to include another representative waveform for control-treated sEPSCs in VTA pDA neurons.

(3) The authors chose to use represent individual data points in Figure 5 and Figure 7, but did include them in all additional figures containing data. The authors should revise their figures to have consistent inclusion or exclusion of individual data points.

Response: We have revised Figures 5 and 7 to include individual data points.

(4) Figure 9 provides a summary of the findings that includes speculation regarding changes in nAChR sensitivity in GABAergic neurons in the VTA. Since this manuscript did not contain inhibitory recordings (or direct imagine evidence) supporting nAChR changes in the VTA, some of the conclusions illustrated in Figure 9 remain somewhat speculative. The authors should consider revising this figure to reflect the need for future work to elucidate the role of farnesene on inhibitory signaling in the VTA.

Response: We have since removed figure 9 as it was a strong assumption based on the data shown here.

(5) Discussion of the implications of translation of these findings to human use (lines 232-233) are somewhat speculative as the current experiments are done in mice. This would be best suited for the Discussion section at the end of the manuscript.

Response: These sentences have been moved to the discussion.
